# Sequential ALK inhibitor treatment benefits patient with leptomeningeal metastasis harboring non‐EML4‐ALK rearrangements detected from cerebrospinal fluid: A case report

**DOI:** 10.1111/1759-7714.13259

**Published:** 2019-11-25

**Authors:** Zhaona Li, Pupu Li, Bing Yan, Qiongqiong Gao, Xiangli Jiang, Zhongli Zhan, Qingna Yan, Analyn Lizaso, Chun Huang

**Affiliations:** ^1^ Tianjin Medical University Cancer Institute & Hospital, National Clinical Research Center for Cancer Tianjin China; ^2^ Key Laboratory of Cancer Prevention and Therapy, Tianjin's Clinical Research Center for Cancer Tianjin China; ^3^ Department of Thoracic Oncology Tianjin Medical University Cancer Institute & Hospital Tianjin China; ^4^ Shenzhen Nanshan People's Hospital Shenzhen China; ^5^ Department of Respiratory Medicine Jinghai Hospital Tianjin China; ^6^ Department of Pathology Tianjin Lung Cancer Center, Tianjin Medical University Cancer Institute & Hospital Tianjin China; ^7^ Burning Rock Biotech Guangzhou China

**Keywords:** Alectinib dose escalation, ALK rearrangement, cerebrospinal fluid, leptomeningeal metastasis, non‐EML4‐ALK

## Abstract

A 47‐year‐old female with ALK‐rearranged lung adenocarcinoma developed leptomeningeal metastasis (LM) after progression on first‐line crizotinib. Alectinib 300 mg was commenced and the patient achieved clinical and radiographic improvements. After nine months of alectinib 300 mg, she started to experience symptomatic LM. Two concurrent non‐EML4‐ALK rearrangements, *LOC388942‐ALK* and *LINC00211‐ALK*, were identified from the CSF but not from the plasma samples. With the primary lung lesions remaining stable, the alectinib dose was increased to 600 mg twice daily which alleviated the clinical symptoms of symptomatic LM. After 7.6 months of alectinib 600 mg, the patient again experienced CNS progression. With both CSF and plasma samples revealing no druggable mutations, the alectinib dose was re‐escalated to 900 mg twice daily, resulting in clinical benefits lasting for two months. Her therapy was then switched to lorlatinib which controlled the disease for 8.7 months until her demise. The *LINC00211‐ALK* fusion, which retains the ALK kinase domain, detected from the CSF was the mechanism of treatment efficacy in this patient. The central nervous system (CNS) has been increasingly recognized as a site of treatment failure in multiple cancers, including non‐small cell lung cancer (NSCLC). Our case demonstrated that alectinib dose‐escalation and lorlatinib overcame ALK inhibitor resistance in the CNS in an ALK‐positive LM patient. Furthermore, we provide the first clinical evidence of the efficacy of sequential ALK inhibitors in targeting *LINC00211‐ALK* in a patient with LM.

## Introduction

Crizotinib, as the first inhibitor targeting the anaplastic lymphoma kinase (ALK) fusion approved by the US Food and Drug Administration, has dramatically benefited non‐small cell lung cancer (NSCLC) patients with ALK rearrangements.^1^ However, crizotinib has an extremely low cerebrospinal fluid (CSF) penetration (0.26%), resulting in a high frequency of recurrences in the central nervous system (CNS).^2^ Despite advances in systemic and local treatment approaches, approximately 4% of molecularly unselected, and 5% to 9% of ALK‐rearranged and EGFR‐mutant NSCLC patients will ultimately develop leptomeningeal metastasis (LM).^3^ LM is defined as the spread of tumor cells into the membranes surrounding the brain, spinal cord, and cranial nerves.^2^ Given the diagnostic challenges, LM is associated with a poor prognosis, with approximately three to six months of median survival in NSCLC patients with LM.^4^ Increasing efforts have been invested in developing next‐generation inhibitors with improved CNS penetration as well as strategies for the detection of LM. Alectinib^5^, ^6^ and lorlatinib,^7^ highly selective second‐generation and third‐generation ALK inhibitors, respectively, showed superior systemic and CNS efficacy as compared with crizotinib.

CSF‐derived circulating tumor DNA (ctDNA) has been demonstrated to be superior to plasma‐derived ctDNA in reflecting mutations in ALK‐rearranged NSCLC patients with LM.^8^, ^9^ The detection of mutations in CSF‐derived ctDNA enables the proper management and improvement in the quality of life in patients with LM. Herein, we describe a patient whose concurrent ALK rearrangements were detected from CSF‐derived ctDNA after development of brain metastasis (BM) and LM and who clinically responded to ALK inhibitors.

## Case report

**Figure 1:**
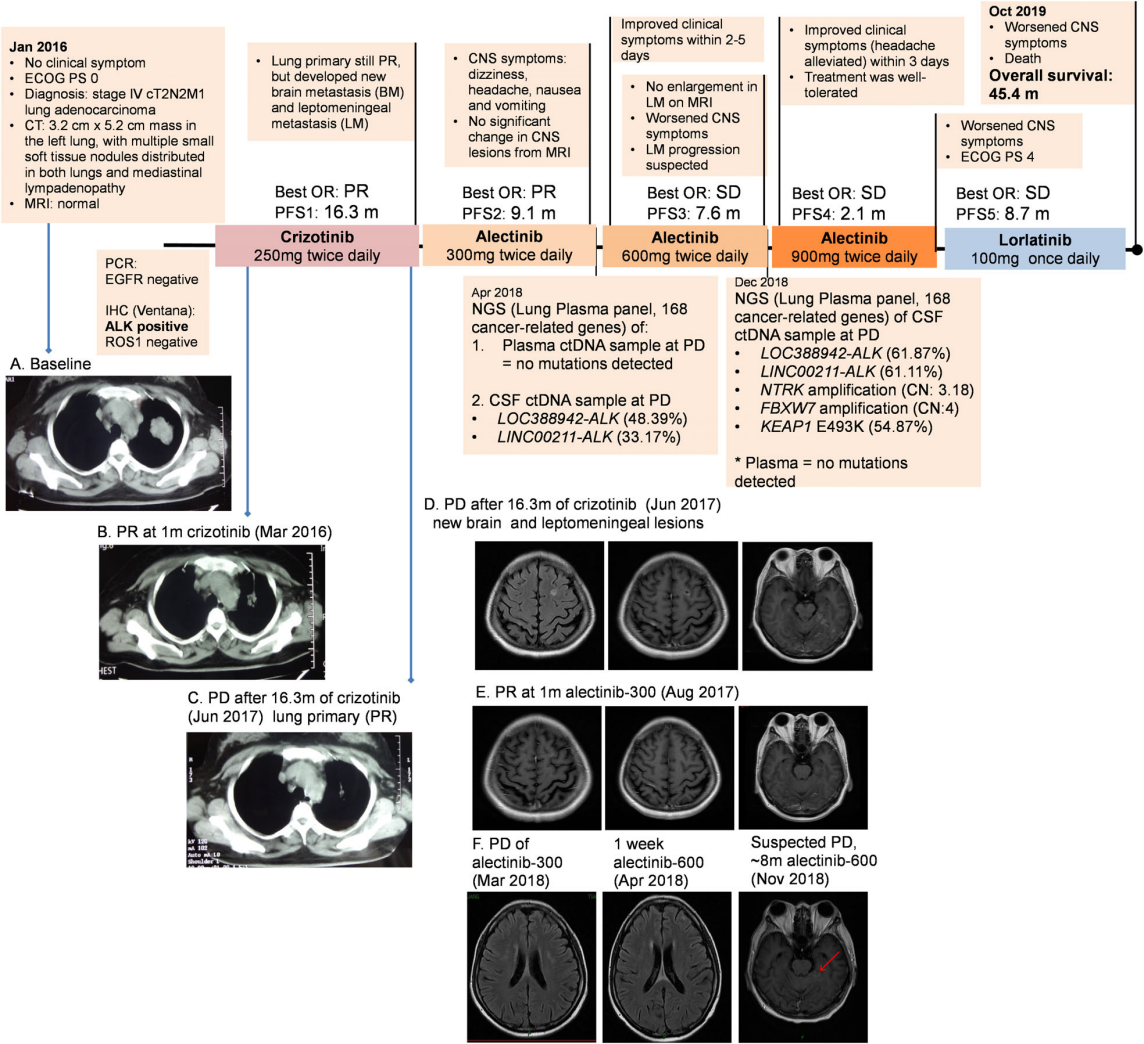
An illustrated summary of the treatment regimen received by the patient including status of clinical symptoms, investigator‐assessed objective responses (OR) based on Response Evaluation Criteria in Solid Tumors (RECIST) v.1.1, progression‐free survival (PFS) (expressed in months [m]) from each line of treatment and the mutations detected with NGS‐based profiling of CSF‐derived ctDNA and plasma‐derived ctDNA samples at indicated time points. Thoracic computed tomography (CT) at (**a**) baseline revealed the 3.2 cm × 5.2 cm mass in the left lung, coupled with multiple small soft tissue nodules distributed in both lungs and mediastinal lymphadenopathy. (**b**) At evaluation of partial response (PR) after one month of crizotinib. (**c**) At 16.3 months of crizotinib, CT scans of the primary lung lesions remained similar as the lesion evaluated at PR. (**d**) However, new metastatic lesions in the brain and meninges were revealed by cranial magnetic resonance imaging (MRI). (**e**) After one month of alectinib therapy at 300 mg, the size of the metastatic lesions in the skull were significantly reduced (0.4 × 0.7 cm), resulting in an evaluation of PR. (**f**) Subsequent MRI showing alectinib treatment course. Best OR, best overall objective response; PD, disease progression; PR, partial response; SD, stable disease.

During a regular health check in January 2016, a 47‐year‐old non‐smoking Chinese woman was diagnosed with stage IV (T2N2M1) lung adenocarcinoma (Fig 1a). Upon detection of ALK rearrangement, crizotinib at 250 mg twice daily (bid) was initiated. Within four weeks of crizotinib therapy, a partial response (PR) was achieved based on the significant reduction in her primary lung lesions (Fig 1b). At 16.3 months of crizotinib therapy, CT scans of the primary lung lesions remained stable, with similar measurements at PR (0.8 × 0.5 cm) (Fig 1c); however, new metastatic lesions in the brain and meninges were revealed through cranial magnetic resonance imaging (MRI) as multiple nodules in the bilateral cerebral hemisphere, with the largest measurable lesion having a diameter of 0.7 × 0.9 cm and abnormally enhanced images in the pia mater and left cerebellum (Fig 1d).

Due to the local unavailability of alectinib in Mainland China, the patient commenced alectinib treatment at 300 mg bid in Japan.^5^, ^6^, ^10^, ^11^ Her dizziness and headache significantly improved within a week of commencing treatment. In addition to an improvement in her clinical symptoms, the brain lesions almost disappeared with the leptomeningeal lesions markedly reduced (0.4 × 0.7 cm) after a month of the alectinib 300 mg bid regimen (Fig 1e). Unfortunately, after 9.1 months of alectinib at 300 mg bid, she again experienced deteriorating dizziness, headache, nausea and vomiting; however, MRI revealed no significant change in CNS lesions. Since LM progression is not radiologically detectable in a fraction of patients, LM progression was suspected based on the clinical symptoms and the progressive increase of intracranial pressure. Interestingly, from the start of alectinib 300 mg bid to the time of LM progression, the lung lesion had remained stable. Capture‐based targeted sequencing of CSF and plasma samples was performed using a panel consisting of 168 cancer‐related genes (Burning Rock Biotech). Analysis revealed the detection of two non‐*EML4‐ALK* fusions in the CSF: *LOC388942‐ALK* and *LINC00211‐ALK*, with an allelic fraction (AF) of 48.39% and 33.17%, respectively. However, no mutation was detected from the plasma. Since no well‐established resistance mechanisms were acquired by the patient during alectinib therapy and the standard dose of alectinib is 600 mg bid outside of Japan,^12^ her alectinib dose was then increased from April 2018. Her clinical symptoms improved within two days and disappeared within five days after the treatment. Although a review of her MRI scan performed seven days after the treatment did not reveal measurable changes (Fig 1f), the marked improvement in clinical symptoms was still considered as treatment efficacy. Despite achieving remarkable improvement in clinical symptoms with alectinib 600 mg bid therapy, the patient experienced worsening of her CNS symptoms at 7.6 months including ptosis of the right eyelid, deteriorating vision of the right‐eye, numbness of the left side of her face, loss of bladder and bowel control, weakness of legs and unsteady gait. Despite the lack of significant enlargement of LM lesions from the MRI, such clinical symptoms are evidence of LM progression. At disease progression, mutation profiling of CSF revealed increased AF for both ALK rearrangements and three additional mutations with no available targeted therapy, while there were still no mutations detected in the plasma sample. She remained on alectinib but with the dose increased to 900 mg bid. After three days of treatment, her headaches were alleviated. However, the clinical benefit only lasted for 2.1 months. Due to worsening of her CNS symptoms, her physical mobility also significantly deteriorated and she was evaluated as having an Eastern Cooperative Oncology Group Performance score of 4. Considering her physical condition, her family refused to grant permission for a lumbar puncture for CSF aspiration to be performed. Based on her consistent treatment response with first‐ and second‐generation ALK inhibitor, a sequential treatment with third‐generation ALK inhibitor was commenced. She was then switched to lorlatinib at a dose of 100 mg once daily. She clinically benefited from lorlatinib therapy for 8.7 months until succumbing to the complications of her disease on 20 October 2019, with an overall survival of 45.4 months from the time of diagnosis.

## Discussion

**Figure 2:**
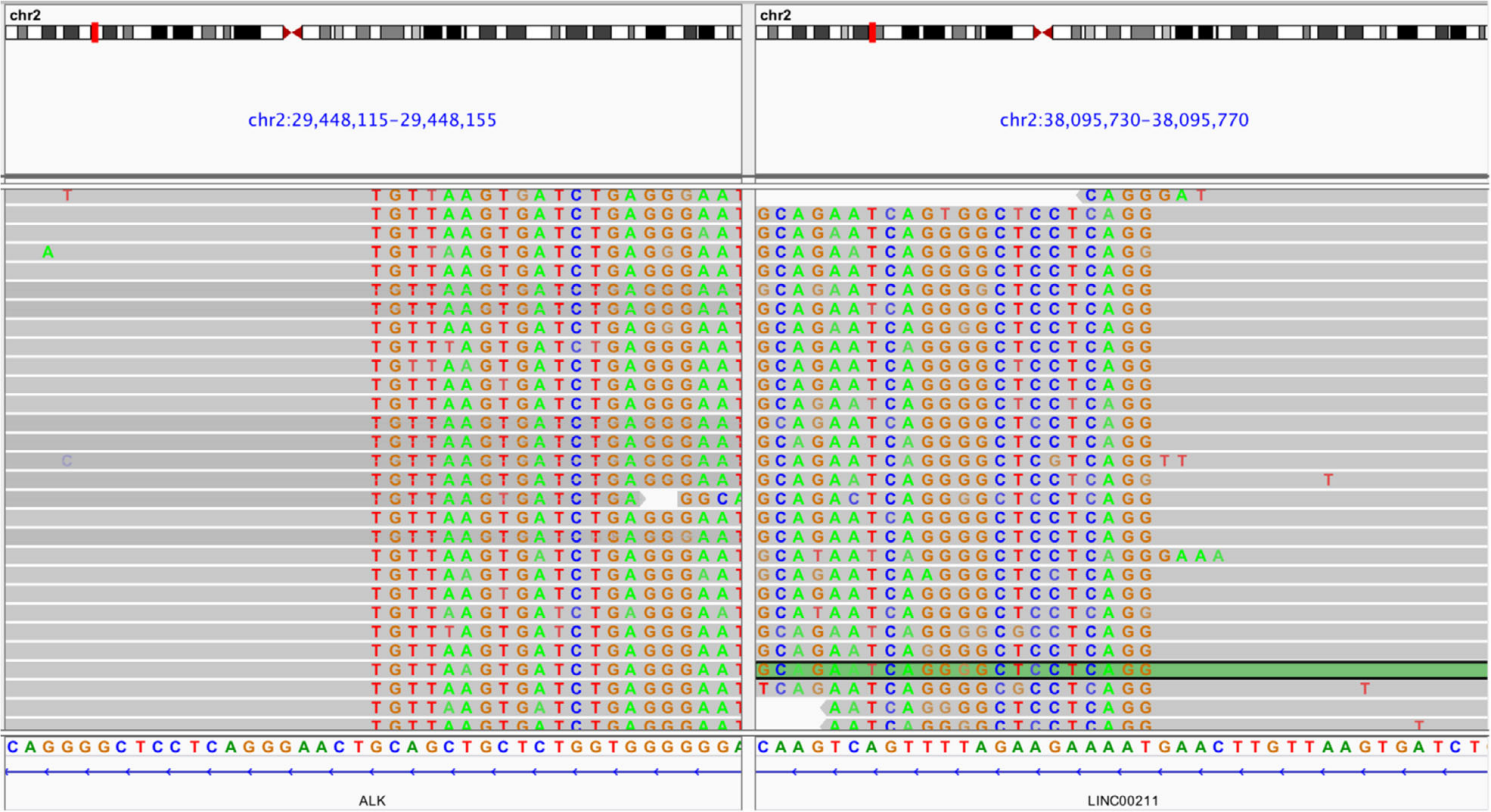
Illustration of the LINC00211‐ALK fusion detected from the CSF sample of the patient at PD from crizotinib therapy. The breakpoints are in chr 2:29448134 for ALK and chr 2:38095752 for LINC00211. Each gray row represents the sequencing read from a DNA fragment. Bottom bar shows the DNA sequence annotation of ALK (left) and LINC00211 (right).

The dosing of targeted therapy typically followed a one‐dose‐fits‐all approach; however, increasing evidence has proven that dose escalation after treatment failure could reverse the resistance in several cancer types including ALK‐rearranged NSCLC.^13^ Dose escalation of alectinib from 600 mg to 900 mg bid has been reported in an ALK‐rearranged NSCLC patient with LM to ensure adequate CNS penetration.^13^ Our patient with LM described in this report benefited from alectinib dose escalation after the detection of two concurrent non‐EML4‐ALK rearrangements from CSF‐derived ctDNA mutation profiling. Owing to the blood‐brain barrier, plasma ctDNA does not fully reflect the mutations in intracranial lesions which poses an obstacle for genomic characterization of CNS lesions.^8^ Since CSF circulates in the brain and spinal cord, it could contain ctDNA and circulating cell‐free DNA (cfDNA) released by CNS tumors. Emerging evidence demonstrated the clinical value of CSF‐based liquid biopsy as a viable specimen for guiding precision treatment, particularly in the diagnosis and treatment monitoring of patients with either primary or metastatic CNS tumors.^8^, ^9^ In our patient, instead of the classic EML4‐ALK rearrangement, two concurrent non‐EML4‐ALK rearrangements previously unreported to respond to alectinib or any other ALK inhibitors were detected in her CSF samples but were not detected in her plasma samples. Further analysis revealed that the ALK kinase domain was not retained in *LOC388942‐ALK*, while *LINC00211‐ALK* has the breakpoint in the intron 19 of ALK and thus retained the critical ALK kinase domain (Fig 2). By collectively considering all the clinical evidence including the stable primary lung lesion, the absence of mutations detected in the plasma samples, the absence of radiologically observable change, the presence of at least one ALK fusion detected from CSF samples potentially responsive to alectinib, the increased intracranial pressure, and the presence of CNS progression‐related clinical symptoms, the care team confirmed the failure of alectinib therapy at 300 mg and decided on escalating the dose to 600 mg and again from 600 mg to 900 mg bid. Increasing the alectinib dosage ensured that adequate concentration penetrated into the CNS thus enabling the reversal of the drug tolerance and prolonging the duration of response to alectinib in our patient. Upon dose escalation, the clinical response of the patient was very apparent within three to five days. This clinical evidence also indicates that ALK signaling, through *LINC00211‐ALK* rearrangement, was a major oncogenic driver of the CNS progression and is responsive to ALK inhibition.

In conclusion, our case demonstrated that alectinib dose‐escalation and lorlatinib were able to overcome ALK inhibitor resistance in the CNS in a non‐EML4‐ALK‐positive LM patient. Furthermore, we provide the first clinical evidence of the efficacy of ALK inhibitors in targeting *LINC00211‐ALK* in a patient with LM.

## Disclosure

A.L. is an employee of Burning Rock Biotech. The other authors declare no competing interests.
